# Human herpesvirus 8-associated colonic Kaposi’s sarcoma during vedolizumab treatment in ulcerative colitis: a case report and review of the literature

**DOI:** 10.1186/s12876-020-01221-2

**Published:** 2020-03-24

**Authors:** Valerio Papa, Maria Cristina Giustiniani, Loris Riccardo Lopetuso, Alfredo Papa

**Affiliations:** 1 Department of Surgery, Fondazione Policlinico A. Gemelli IRCCS, Rome, Italy; 2 Istituto di Clinica Chirurgica, Università Cattolica del S.Cuore, Rome, Italy; 3 Department of Surgery, Ospedale S. Carlo di Nancy, Rome, Italy; 4 Department of Pathology, Fondazione Policlinico A. Gemelli IRCCS, Rome, Italy; 5 UOC Medicina Interna e Gastroenterologia, Area Gastroenterologia ed Oncologia Medica, Dipartimento di Scienze Gastroenterologiche, Endocrino-Metaboliche e Nefro-Urologiche, Fondazione Policlinico Universitario A. Gemelli IRCCS, L.go A. Gemelli 8, 00168 Rome, Italy; 6 Istituto di Patologia Speciale Medica, Università Cattolica del Sacro Cuore, Rome, Italy

**Keywords:** Ulcerative colitis, Kaposi’s sarcoma, Vedolizumab, Human Herpesvirus-8

## Abstract

**Background:**

Kaposi’s sarcoma (KS) is a rare vascular tumor associated with human herpesvirus (HHV)-8 infection. One of the variants of KS is defined iatrogenic and is overall reported in transplanted patient but also, although less frequently, in patients treated with long-standing immunosuppressive therapy, such as in inflammatory bowel disease including ulcerative colitis and Crohn’s disease.

**Case presentation:**

Herein, we report the first case of KS in a human immunodeficiency virus (HIV)-negative 47-year old male with UC after treatment with the α4-β7 integrin inhibitor vedolizumab (VDZ). The patient underwent to colectomy for a medical refractory disease and the histological examination of the surgical specimen showed the typical findings of KS together with the HHV-8 positivity. The patient achieved a good health status, without any sign of disease recurrence.

**Conclusions:**

In the present case, we can assume that VDZ may have promoted the reactivation of a latent HHV-8 infection endowed with oncogenic potentialities and, in turn, the onset of KS. We also briefly reviewed all the cases of KS in HIV-negative patients with inflammatory bowel disease.

## Background

Kaposi’s sarcoma (KS) is a rare vascular tumor associated with human herpesvirus (HHV)-8 infection [1]. KS lesions typically involve the skin or mucosal surfaces and are characterized by multiple red-purple or brown-black macules, papules, and nodules [2]. Definitive diagnosis requires histologic examination revealing peculiar angio-proliferative features with the typical spindle cell proliferation [1–3]. There are four recognized epidemiologic-clinical types of KS, which are histologically indistinguishable: classic, endemic (African), epidemic (acquired immunedeficiency syndrome-associated) and iatrogenic (immunosuppressive therapy-related) [1]. Iatrogenic KS has been described overall in transplanted patient but has also less frequently been reported in other categories of patients who underwent to long-standing immunosuppressive treatment, such as patients with inflammatory bowel disease including ulcerative colitis (UC) and Crohn’s disease (CD) [2, 3].

## Case presentation

**Fig. 1:**
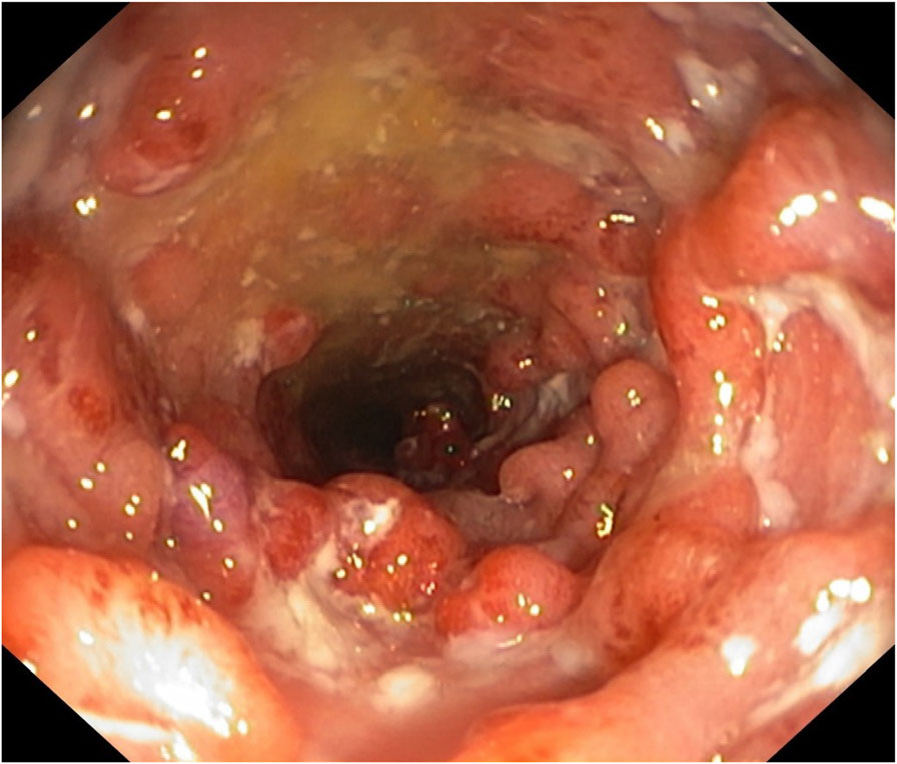
Endoscopic aspects of the transverse colon before colectomy. There are diffuse mucosal ulcerations and nodules/pseudopolyps

**Fig. 2:**
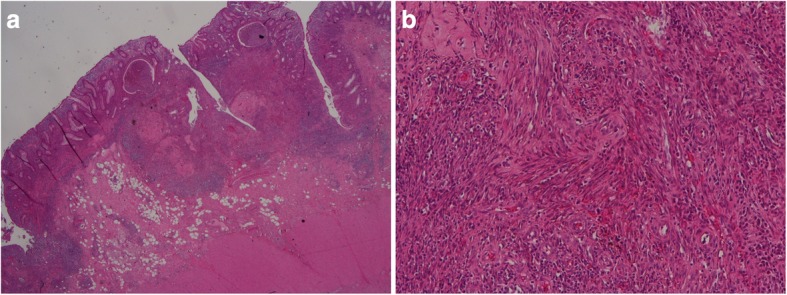
**a** Histologic examination of the colonic surgical specimen (hematoxylin-eosin 1x)**:** spindle cells submucosal nodular proliferation. **b** Histologic examination of the colonic surgical specimen (Hematoxylin –eosin 4x): spindle cells-shaped cells forming vascular spaces with red blood cells percolating between them

**Fig. 3:**
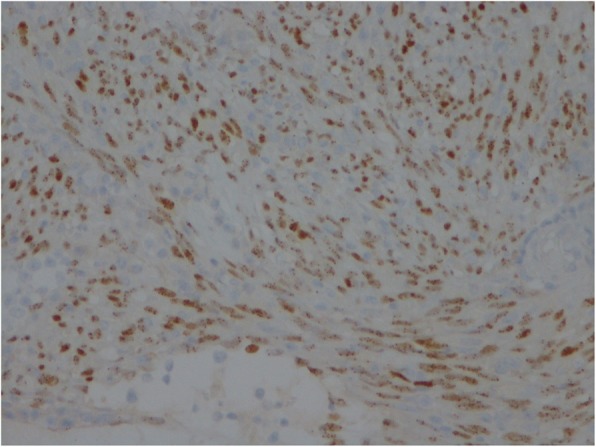
Spindle cells stained with HHV-8

Herein, we report a case of a 47-year-old heterosexual Caucasian man, who was diagnosed with UC in 2010. At the onset, the disease was localized to the entire colon and the patient was initially treated with oral prednisone and then with mesalazine for about 3 years. In 2013, following a severe relapse with partial response to steroids he started infliximab (IFX) at standard dosage (5 mg/Kg) and achieved a clinical and endoscopic remission. IFX was maintained every 8 weeks until July 2017 when it was withdrawn for a progressive loss of response. At September 2017, the patient experienced a disease flare characterized by 5–6 bowel movements/day of liquid and bloody stools and abdominal pain. Partial Mayo index score was 7 (7–9 indicates severe disease). Laboratory exams revealed anemia (hemoglobin 10.3 g/L) and elevated C-reactive protein (18 mg/L, normal value < 5). So, after the exclusion of intestinal infections, the α4-β7 integrin inhibitor vedolizumab (VDZ) was started at the standard dosage of 300 mg (intravenous infusion). VDZ was administered at time 0, 2 and 6 weeks following the standard induction protocol. The patient reported an early clinical benefit and thus received other three administrations every 8 weeks. Nevertheless, he showed a progressive loss of clinical response and consequently VDZ was discontinued. The patient underwent to a colonoscopy in April 2018, which showed a severe pancolitis with deep ulceration, spontaneous bleeding and nodular mucosa particularly in the transverse and in the right colon (Mayo endoscopic score 3) (Fig. 1). Rectal biopsies were taken. Histopathological evaluation of intranuclear cytomegalovirus inclusions resulted negative. Finally, a laparoscopic colectomy with temporary ileostomy was performed. Surprisingly, histological examination of the surgical specimen at level of rectal mucosa and submucosa showed a spindle cells submucosal nodular proliferation suggestive of KS (Fig. 2a-b) and subsequent immunohistochemical staining detected spindle cells stained with HHV-8 (Fig. 3) confirming the diagnosis of colonic KS. HIV serology resulted negative and skin examination and upper digestive tract endoscopy were normal. Four months after the colectomy, the patient underwent to ileal pouch-anal anastomosis surgery. At present, the patient shows an overall good health state without any sign of KS recurrence.

## Discussion and conclusions

Immunosuppression plays a crucial role in KS pathogenesis by stimulating HHV-8 proliferation and, in turn, proto-oncogene expression [1–3]. In most cases, iatrogenic KS appears in kidney-transplanted patients. However, it has also been reported in chronic inflammatory conditions that require immunosuppressive therapies for a long period such as UC [4–20] and CD [21–24]. In this scenario, the anti-TNF-α agents, the anti-integrin agent VDZ and, recently, the anti-interleukin (IL)-12/23 ustekinumab have been added to the IBD therapeutic armamentarium, which already included steroids and the traditional immunosuppressants (i.e., azathioprine, methotrexate, and cyclosporine). All these biologics, as easily predictable, carry out a variable increased risk of opportunistic infection. Overall, 21 cases of KS in HIV-negative patients with IBD are reported. Most of them are adult males with UC refractory to medical therapy, who assumed steroids and/or traditional immunosuppressants at the time of diagnosis and did not show any cutaneous lesions (Table 1). However, in the last years, some cases of KS have been reported in patients treated with IFX and, as far as we know, the present case is the first associated to the administration of the humanized monoclonal antibody VDZ. Indeed, herein we report an HHV-8-associated colonic KS in a patient with UC treated for 8 months with VDZ. Although we cannot rule out the role of previous treatments with steroids and especially with IFX to which the patient had previously undergone in promote the reactivation of HHV-8.

**Table 1:** Characteristics of HIV-negative patients with inflammatory bowel disease (IBD) and intestinal Kaposi’s sarcoma (KS)

Author (References)	Sex	Age yrs	Type of IBD	Disease duration yrs	Immuno-suppressive therapy	HHV-8 in colon	Skin involvement	Colectomy or small bowel resection
Pioche M [11]	M	49	UC	2	CS/AZA/CYCL/IFX	+	–	+
Rodriquez-Pelaez M [13]	M	65	UC	15	CS/MTX	+	+	+
Herculano R [15]	M	63	UC	< 1	CS	+	–	–
Kumar V [16]	M	70	UC	4	CS/IFX (1 infusion)	+	–	+
Hamzaoui L [14]	M	30	UC	2	CS/AZA/IFX	+	–	+
Bursics A [8]	M	49	UC	5	CS	–	+	+
Duh E [17]	M	48	UC	25	CS/AZA	+	–	+
Carmo J [18]	M	58	UC	NA	CS	NA	–	–
Girelli GM [10]	M	43	UC	< 1	CS/CYCL	+	–	+
Cetin B [12]	M	42	UC	13	CS/AZA	–	+	–
Thompson (1989)	M	23	UC	1	CS	NA	–	+
Svrcek M [9]	M	62	UC	30	CS/AZA	+	–	+
Tedesco M [7]	M	68	UC	8	CS	NA	–	+
Meltzer SJ [4]	M	83	UC	< 1	CS	NA	+	+
Pedulla F [5]	M	35	UC	2	CS/AZA	NA	–	NA
Chtourou L [19]	M	53	UC	< 1	CS/AZA	+	–	+
Shah N [20]	M	49	UC	NA	CS/AZA	+	–	+
Koop HO [21]			CD		CS	NA	+	+
Puy-Montbrun T [22]	F	36	CD	NA	CS/AZA	NA	–	–
Cohen RL [23]	F	67	CD	25	CS	NA	–	+
Windon AL [24]	M	21	CD	1	CS/IFX	–	–	+

*UC* ulcerative colitis, *CD* Crohn’s disease, *CS* corticosteroids, *AZA* azathioprine, *CYCL* cyclosporine, *MTX* methotrexate, *IFX* infliximab, *VDZ* vedolizumab, *HHV-8* human herpes virus-8, *NA* not available

VDZ selectively targets the α4-β7 integrin that binds to mucosal addressin-cell adhesion molecule-1 (MadCAM-1) to mediate T cell homing to the lamina propria of the small intestine [25, 26]. VDZ has been approved for the treatment of moderately to severely active UC and CD in adults who failed to respond to at least one conventional drug. Differently from the other available anti-integrin agent Natalizumab (approved in the United States and Europe as monotherapy for multiple sclerosis and only available through a specific risk-minimization program), VDZ selectively acts at intestinal level in order to avoid the risk of progressive multifocal leukoencephalopathy (PME) that due to the reactivation of JC polyomavirus (JCV) [27]. A recent systematic review on VDZ safety profile included data not only from registration studies but also from real life experiences and concluded that overall data are insufficient to draw definitive conclusions about the risk of malignancy linked to VDZ. Indeed, a reduction in immuno-surveillance, as a consequence of leucocyte trafficking inhibition, represents a theoretical concern for gastrointestinal malignancies [26]. In the future, we will definitely assess an increasing number of IBD patients treated in sequence with different biological and immunosuppressive drugs (as in this case-report). Thus, the number of neoplasms, such as KS, linked to a state of immunosuppression that allows a reactivation of latent oncogenic viruses together with a reduced local immuno-surveillance will probably become a more frequent problem. Luckily, all cases of KS occurred in HIV-negative IBD patients, resolved with the discontinuation of immunosuppressive therapy and with colectomy or resection of the affected intestinal tract. In conclusion, this and the other cases described should alert clinicians regarding the possibility of the occurrence of colonic KS in patients with IBD (particularly UC) refractory to medical therapy and who have been treated for a long-time with several immunosuppressive and biological drugs. For the first time we described a case of VDZ-associated colonic KS. VDZ thanks to its specificity of action at the intestinal level may cause the reactivation of latent HHV-8 infection with a consequent initiation of the oncogenic processes that can lead to the onset of KS. Unfortunately, at the moment we do not have reliable tests to identify patients at increased risk of developing KS (there are few data on the usefulness of a specific PCR for the detection of HHV-8 in the blood) that should be recommended for early surgery rather than other rescue therapy. Therefore, further studies are necessary to identify early risk markers of intestinal KS. In the meantime, careful monitoring is required.

## Data Availability

All data generated or analyzed in this manuscript are included in this published article.

## Abbreviations

KS: Kaposi’s sarcoma
HHV: Human herpesvirus
UC: Ulcerative colitis
HIV: Human immunodeficiency virus
CD: Crohn’s disease
IFX: Infliximab
MadCAM-1: Mucosal addressin-cell adhesion molecule-1
PME: Progressive multifocal leukoencephalopathy
JCV: JC polyomavirus
